# Satisfaction Level among Patients Treated with Fixed Dental Prosthesis in a Tertiary Care Hospital: A Descriptive Cross-sectional Study

**DOI:** 10.31729/jnma.4720

**Published:** 2020-01-31

**Authors:** Lajana Shrestha, Sirjana Dahal, Dilesh Pradhan, Junu Lohani

**Affiliations:** 1 Department of Prosthodontics and Maxillofacial Prosthetics, Kathmandu Medical College, Duwakot, Bhaktapur, Nepal; 2 Department of Community and Public Health Dentistry, Kathmandu Medical College, Duwakot, Bhaktapur, Nepal

**Keywords:** *dental prosthesis*, *interdental aids*, *satisfaction*

## Abstract

**Introduction:**

Dental treatment aims at correction of existing disease; prevention of future disease with rehabilitation of patient's lost functional capacity and aesthetics. Fixed dental prosthesis is any prosthesis that is cemented to a natural tooth or dental implants abutments that cannot be removed by patient. The success of prosthodontic treatment is related to prosthesis survival, with its ability to fulfil biologic and patient-evaluated objectives with patient satisfaction. This study is aimed to find the patient satisfaction with fixed prosthodontic treatment.

**Methods:**

This descriptive cross-sectional study was done in a tertiary care hospital among 102 patients rehabilitated with fixed dental prosthesis from August to September 2019 after taking ethical approval from Institutional Review Committee of Kathmandu Medical College. (IRC No. 1207201918). Convenience sampling was done. The questionnaire assessed patient's satisfaction of fixed prosthesis on the basis of appearance, chewing ability, cleansibility, speech and awareness of oral hygiene measures for cleaning of the prosthesis. Data entry was done in Microsoft excel and analysed using Statistical Package for Social Sciences (SPSS)version 20.0, point estimate at 95% Confidence Interval was calculated along with frequency and proportion for binary data.

**Results:**

The majority of the patients 87 (85.3%) were satisfied with their fixed prosthesis, at 95% confidence interval (93.5-81%). Eighty one (79.4%) were satisfied with their chewing ability; 99 (97.1%) satisfied with their speech, 78 (76.4%) satisfied with appearance of fixed prosthesis. Ninety eight patients (96.1%) were aware of oral hygiene measures, out of which only 66 (67.3%) used interdental aids for cleaning of their fixed prosthesis.

**Conclusions:**

Several factors (chewing ability, appearance, speech, cleansibility of fixed prosthesis) had positive impact on overall satisfaction in majority of the patients. Dentists should continue to emphasise on the significance of maintaining good oral hygiene and use of interdental aids for the longevity of fixed prosthesis.

## INTRODUCTION

Teeth do not possess regenerative ability as found in most other tissues. So, tooth structure lost due to caries, periodontal diseases, and trauma are restored by direct restoration or by restoration fabricated outside mouth. Depending upon patients' oral health conditions, there are removable and fixed treatment methods of rehabilitation.^1^

Fixed prosthodontic treatment involves replacement and restoration of teeth by artificial substitutes that are not readily removed from mouth.^2^ It will improve patient comfort and masticatory ability, maintain health and integrity of dental arches, and elevate patient's self-image.^3^ The success of prosthodontic treatment is related to prosthesis survival with its ability to fulfill biologic related and patient-evaluated objectives along with patient satisfaction.^4,5^ To authors' knowledge, literature regarding assessment of patients' satisfaction treated with fixed dental prosthesis in Nepal is scarce.

This study aimed to find the patient satisfaction with fixed prosthodontic treatment and assess oral hygiene practice awareness.

## METHODS

This descriptive cross-sectional study was carried out among patients rehabilitated with fixed dental prosthesis at Kathmandu Medical College, Duwakot for two months from August to September 2019.

Ethical approval for the study was obtained from Institutional Review Committee of Kathmandu Medical College (IRC No. 1207201918). Male and female patients who had received any form of fixed dental prosthesis at Department of Prosthodontics, Kathmandu Medical College and those who were willing to sign written consent were included in the study. The privacy of the participants was fully maintained and written consent was obtained after explaining in detail the entire study protocol. Those who did not give consent for any reason were excluded from the study. No names, documents or results are disclosed or circulated anywhere other than among the researchers. The names of the participants do not appear in the final report.

Convenience (non-probability) sampling method was utilised and the sample size of 94 was calculated by using following formula:

$$\begin{array}{ll} \text{n} & \text{= }{\text{Z}}^{\text{2}}\times (\text{p}\times \text{ q)}/{\text{e}}^{2}\\ & \text{= 1}{\text{.96}}^{\text{2}}\times \text{0.43}\times \text{(1}-\text{0.43)}/\text{0}{\text{.11}}^{\text{2}}\\ & \text{= }0.94/0.0121\\ & =77.68\\ & =78 \end{array}$$

Where,

n = required sample size
p = prevalence of condition^6^
q = 1-p,
e = margin of error, 11%
Adding 20% of non-response (94 patients)

However the total sample taken was 102 after adding 20% of non-response (98 patients). Based on the relevance to the population, we adapted the previously used questionnaires to our study setting.^5,6^ The questionnaire was developed to assess patient's satisfaction of fixed dental prosthesis on the basis of appearance, chewing ability, cleans ability and speech. The study also aimed to assess awareness of oral hygiene measures, use of aids for cleaning of fixed dental prosthesis. Patients were interviewed and the data obtained were subsequently entered Microsoft excel sheet and analysed using Statistical Package for Social Sciences (SPSS) version 20.0, point estimate at 95% Confidence Interval was calculated along with frequency and proportion for binary data.

## RESULTS

Out of 102 participants enrolled in the study, 87 (85.3%) were in overall satisfied category with the fixed prosthesis at 95% confidence interval (93.5-81%) (Table 1).

**Table 1:** Satisfaction of fixed prosthesis.

1. How satisfied are you with appearance of your fixed prosthesis?	
Satisfied	78 (76.4)
Neutral	15 (14.7)
Unsatisfied	9 (8.8)
2. How satisfied are you with the cleansibility of your fixed prosthesis?	
Satisfied	98 (96.1)
Neutral	1 (1.0)
Unsatisfied	3 (2.9)
3. How satisfied are you with the chewing ability of fixed prosthesis?	
Satisfied	81 (79.4)
Neutral	8 (7.8)
Unsatisfied	13 (12.7)
4. How satisfied are you with your fixed prosthesis regarding the speech?	
Satisfied	99 (97.1)
Neutral	0
Unsatisfied	3 (2.9)
5. Over-all, how satisfied are you with your fixed prosthesis?	
Satisfied	87 (85.3)
Neutral	10 (9.8)
Unsatisfied	5 (4.9)

Among 102 participants, 47 were males with mean age of 38.4±13.33 years and 55 were females with mean age of 40.47±12.08 years. Among the participants 51 (50%) were university educated (Table 2).

**Table 2:** Gender- wise and education-wise distribution of patients.

Level of education	Gender of patient		n (%)
	Male n (%)	Female n(%)	
Primary	4 (3.9)	7 (6.9)	11 (10.8)
Secondary	7 (6.9)	11 (10.8)	18 (17.6)
Higher education	4 (3.9)	12 (11.8)	16 (15.7)
University	31 (30.4)	20 (19.6)	51 (50.0)
Others	1 (1.0)	5 (4.9)	6 (5.9)
Total	47 (46.1)	55 (53.9)	102 (100)

**Figure 1.**
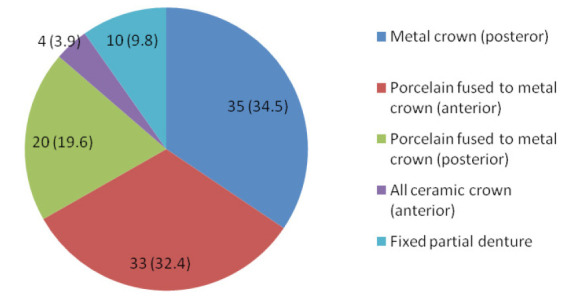
Types of fixed prosthesis.

Fixed prosthesis delivered, included 55 posterior crowns, 37 anterior crowns and 10 fixed partial dentures (Figure 1). The mean duration of placement of fixed prosthesis was 1.57±0.85 years.

Forty two (41.2%) patients occasionally had problem of food getting stuck between fixed prosthesis, when eating fibrous food like meat, green leafy vegetables etc., whereas 56 (54.9%) never had such problem. Only 11 (10.8%) patients complained of bad smell sometimes after placement of fixed prosthesis, whereas 79 (77.5%), never had problem of bad smell after placement of fixed prosthesis.

Regarding oral hygiene awareness, 98 (96.1%) patients were aware of oral hygiene measures, out of which only 66 (67.3%) used interdental aids for cleaning of the fixed prosthesis. The frequency of the use of different interdental aids and the source of information about the use of interdental aids for cleaning of the prosthesis was calculated (Table 3).

**Table 3:** Interdental aids for cleaning of prosthesis and sources of information.

n (%)		
Interdental Dental aids	Dental floss	60 (90.9)
	Interdental brush	16 (24.2)
	Others (toothpick)	3 (4.5)
Source of information about dental aids	Dentist	52 (78.8)
	Personal communication	13 (19.7)
	Others	2 (3.0)

Out of 32 (32.7%) patients not using interdental aids for cleaning of fixed prosthesis, 17 (53.1%) were not informed by the treating dentist about the need of interdental aids for cleaning the prosthesis. Others had difficulty in approaching the fixed prosthesis area and lack of availability of dental aids. Majority of the patients 87 (85.3%) will recommend the treatment with fixed prosthesis to others as well and only 4 (3.9%) said they will never recommend such treatment to others.

## DISCUSSION

The performance of any fixed prosthesis is evaluated by measuring subjective patient-based outcomes of appearance, function and longevity and technical aspects. While in the present study, only the patient based measurement was implied and clinician aspect was not explored. Anderson asserted that the level of satisfaction of both the clinician and patient have to be taken into consideration.^7^ However, many researchers found that the level of patients' satisfaction exceeded that of their dentists.^8–12^ This finding may be due to differences in the criteria used for evaluation by the clinician and the patient. The clinicians' evaluation mainly focuses on the technical characteristics of the prosthesis, while the patients' evaluations in based on subjective criteria like appearance, function and comfort of the prosthesis.^13^

Regarding over-all satisfaction, 87 (85.3%) were satisfied with fixed prosthesis showing positive impact of prosthesis on the patients' oral health. The overall satisfaction positively influenced the patients in recommending similar treatment to others. In the study by Kashbur et al,^14^ 80.9% patients were over all satisfied with the fixed prosthodontic treatment. Tan et al.^15^ observed very high levels of satisfaction in relation to functional aspects of fixed prosthesis like aesthetics, mastication, speech and comfort levels. Kola et al.^6^ reported high level of satisfaction in patients' undergone fixed prosthodontic treatment. In the study by Zavanelli et al.^16^ most (72.58%) of the patients were satisfied with their fixed prosthesis. Good patient satisfaction was also found in an 18-year retrospective study by Napankangas et al.^17^

In the present study only 78 (76.4%) patients were satisfied with the appearance of fixed prosthesis. Nine (8.8%) were unsatisfied due to colour mismatch with natural teeth, mismatch of shape and size of fixed prosthesis compared to natural teeth. The result is comparable to other studies conducted by Geiballa et al,^18^ in which 80% of the participants were pleased with the aesthetic outcome of fixed prosthesis and in study by Kashbur et al.^14^ 14.7% of the patients found their fixed restoration aesthetically unpleasant. Patients' acceptance of fixed also depends upon patients' oral perception and ability to distinguish outer contour of fixed prosthesis. Agrawal et al.^19^ in their study concluded that patients' acceptance of fixed prosthesis not only depends on dentists' procedure of treatment and patients' perception of functional ability and appearance, but also depends on patients oral interpretation and discriminatory skill for external contour of fixed prosthesis.

In the present study 81 (79.4%) were satisfied with chewing ability with fixed prosthesis. In the study conducted by Kola et al.^6^ 59 (39.33%) patients were satisfied with the masticatory ability of fixed prosthesis. Similarly in study by Geiballa et al.^18^ found that 46.4% of the patients felt more comfortable with their fixed prosthesis. Kashbur et al.^14^ found the 21.6% of the patients were not contented with the masticatory function of fixed prosthesis. Geiballa et al.^18^ reported that more than 90% of their individuals had no phonetic alteration with FPD. In the study by Kashbur et al,^14^ almost two thirds of the participants reported altered phonetics after having fixed prosthesis. In the present study 97.1% of the patients had no problems with speech after placement of fixed prosthesis.

Our patient pool shows almost similar numbers of male and female patients, with slightly greater number of female patients. The finding is in contrast to the findings found in other studies, where the female participants were more than double than male participants.^5,14,18^ This may be because of increased awareness among male patients regarding the need of prosthodontic rehabilitation of their mutilated dentition and are becoming more conscious about their appearance.

Maintenance of good oral hygiene is very important to avoid periodontal problems and prevent development of carious lesion. Majority of the patients were aware of oral hygiene measures (98,96.1 %) out of which 66 (67.3%) used interdental aids for cleaning of the fixed prosthesis. This finding suggests that the patients were more aware of and practice oral hygiene measures after placement of fixed prosthesis compared to study by Geiballa et al.^18^ where majority of their patients (94%) did not practice oral hygiene measures after fixed prosthodontic treatment. This satisfactory result may be related to post treatment oral hygiene instruction explained by the dentists; dentists being the major source of information 52 (78.8%) about need of use of interdental aids after fixed prosthodontic treatment. However, this assessment was only based on patients' perception of performing proper oral hygiene practice without undertaking clinical evaluation. It would be useful if clinical evaluation was performed to this group to compare between patients use of interdental aids and maintenance of good oral hygiene. However, those not using dental aids said that the reason for not using the same was that they were not informed by the treating dentist 17 (53.1%) about the need of interdental aids for cleaning the prosthesis and others gave the reasons of difficulty to approach the fixed prosthesis and lack of availability of interdental aids. This result shows that dentists play major role in motivating patients in maintenance of good oral hygiene. The study by Roscher et al.^20^ concluded that professional advice and instruction and reinstruction seemed very important in order to obtain good plaque control around fixed dental prosthesis.

## CONCLUSIONS

The patients with fixed dental prosthesis showed high level of satisfaction in aesthetics and function with positive impact on overall satisfaction and motivating others in receiving similar treatment options. However, the present study is based on patient perceived measurement without clinical evaluation. Maintenance of good oral hygiene with the use of interdental aids is very important for long term success of fixed prosthodontic treatment. Dentists have to pay attention to the post-treatment instructions concerning the maintenance of fixed prosthesis.

## Conflict of Interest

**None.**
